# Editorial: Rehabilitation interventions for mild traumatic brain injury

**DOI:** 10.3389/fneur.2025.1637653

**Published:** 2025-06-24

**Authors:** Fahim Anwar

**Affiliations:** Cambridge University Hospital NHS Foundation Trust, Cambridge, United Kingdom

**Keywords:** brain, injury, mild, rehabilitation, traumatic

Traumatic brain injury is a significant cause of mortality and morbidity worldwide. Traditionally, traumatic brain injuries are classified as mild, moderate, or severe in nature. Mild traumatic brain injury (mTBI) is the most common injury compared to moderate and severe injuries. The recovery pattern from mTBI is highly heterogeneous, with many patients recovering within days to weeks. However, a significant proportion of patients with mTBI will experience a persistent constellation of symptoms ranging from physical and cognitive to vestibular and emotional. The variability in outcomes after mTBI reinforces the importance of early assessment and tailored rehabilitation to reduce the impact of their symptoms on the overall quality of life. It also emphasizes the need for ongoing research and innovative rehabilitation interventions to address the challenges faced by these individuals, which in some cases, could be lifelong.

The Research Topic “Rehabilitation Interventions for Mild Traumatic Brain Injury” has brought together five articles with diverse and innovative topics related to rehabilitation interventions for the multifaceted challenges of mTBI. These topics range from symptom burden and functional connectivity to novel neurostimulation approaches and the culturally integrative and holistic management of mTBI.

In their study, Mercier et al. evaluated a 12-week aerobic exercise intervention in adults with persisting post-concussion symptoms. The study included 50 adult mTBI patients who completed a 12-week sub-symptom threshold aerobic exercise program. The study provides compelling evidence that individualized aerobic activity is associated with reduced symptom burden, improvement in overall quality of life, and improvement in exercise tolerance and dizziness, even in patients with long-standing exercise intolerance. However, the study also showed that there was no change in autonomic markers such as heart rate variability. This suggests that symptom improvements were primarily due to neuroplasticity rather than direct autonomic recalibration.

In a case study exploring the rapidly developing field of neuromodulation, Riccitelli et al. used focal-coil transcranial magnetic stimulation (rTMS) combining inhibitory stimulation on the right dorsolateral prefrontal cortex with excitatory stimulation on the left dorsolateral prefrontal cortex in a 34-year-old woman with severe traumatic brain injury and complex psychopathology. After 2 weeks of treatment, the patient showed decreased impulsivity and obsessive-compulsive symptoms along with improved attention and processing speed. There was a further reduction in impulsivity at 4 weeks with a persistent positive effect observed at 8 weeks. These findings suggest the importance of managing the psychological effects of traumatic brain injury with novel techniques and warrant further exploration in clinical trials.

A similar innovative approach was described by Chu et al. an experimental device was used to deliver a translingual neural stimulation (TLNS) during focused physical therapy sessions for 2 weeks in patients with mTBI to improve gait and balance. Resting-state fMRIs were used pre- and post-intervention to collect datasets. The study found that TLNS was an effective tool in increasing somatosensory processing, vestibular-visual interactions, control and flexible shifting. The authors suggested that TLNS could be an effective approach to induce brain plasticity and could serve as a potential therapy for mTBI for improving gait and balance-related deficits in the future in this population.

In addition to innovative techniques, this Research Topic also includes a systematic review and meta-analysis by Li et al. evaluating the synergistic effect of acupuncture combined with hyperbaric oxygen therapy in patients with TBI. This review included 11 randomized controlled trials involving 896 participants and found that combination therapy significantly improved Glasgow Coma Scale (GCS) scores and consciousness recovery, and that the overall treatment was more effective compared to hyperbaric oxygen alone. These findings highlight the importance of integrating traditional therapeutic modalities with contemporary neurorehabilitation approaches to optimize outcomes in different clinical settings.

Finally, Wu et al. conducted a single-center randomized controlled trial investigating the perioperative use of hydrogen inhalation in patients undergoing glioma surgery, which has implications for TBI-related brain edema. Although glioma surgery lies outside the strict mTBI domain, this study provides characteristic insights into the neuroprotective properties of hydrogen inhalation for managing brain edema due to secondary brain injury, particularly as a non-invasive and anti-inflammatory intervention. Hydrogen inhalation significantly reduced brain edema and improved postoperative sleep and pain scores. These findings suggest a potential use of the technique in mild to moderate TBI to reduce edema and prevent secondary brain damage.

Overall, this Research Topic has examined the evolving landscape of rehabilitation interventions following mTBI. The studies have focused not only on the complexity of the symptoms but also on the potential for recovery through the patient-centered conventional and non-conventional interventions. As the topic editor, I commend all the authors for the methodological robustness and innovative ideas of their studies, along with the application of these ideas to improve the quality of life of patients with mTBI and diverse symptoms. These studies highlight that there is no one-size-fits-all solution for patients with mTBI but rather an adaptive rehabilitation process informed by neurobiology, function, and lived experience. Future research should aim to refine treatment algorithms, personalize interventions, and ultimately bridge the gap between emerging evidence and routine clinical practice.

